# Disease management for co-morbid depression and anxiety in diabetes mellitus: design of a randomised controlled trial in primary care

**DOI:** 10.1186/1471-2296-12-139

**Published:** 2011-12-15

**Authors:** Corinne H Stoop, Viola RM Spek, Victor JM Pop, François Pouwer

**Affiliations:** 1 Department of Medical Psychology & Neuropsychology, Center of Research on Psychology in Somatic diseases (CoRPS), Tilburg University, Tilburg, The Netherlands

**Keywords:** diabetes, depression, anxiety, primary care, study protocol, randomised controlled trial, disease management, stepped care, psychological intervention

## Abstract

**Background:**

Depression and anxiety are common co-morbid health problems in patients with type 2 diabetes. Both depression and anxiety are associated with poor glycaemic control and increased risk of poor vascular outcomes and higher mortality rates. Results of previous studies have shown that in clinical practice, treatment of depression and anxiety is far from optimal as these symptoms are frequently overlooked and undertreated.

**Methods/Design:**

This randomised controlled trial will examine the effectiveness of a disease management programme treating symptoms of depression and anxiety in primary care patients with Type 2 diabetes. Patients will be randomized on patient level in 1:1 ratio. Random block sizes of 2 and 4 are used. The disease management programme consists of screening, stepped treatment and monitoring of symptoms (n = 80). This will be compared to care as usual (n = 80).

**Discussion:**

The disease management model for co-morbid depression and anxiety in primary care patients with diabetes is expected to result in reduced symptoms of depression and anxiety, improved quality of life, reduced diabetes specific distress and improved glyceamic control, compared to care as usual.

**Trial Registration:**

Dutch Trial Register NTR2626

## Background

Diabetes mellitus is a common chronic disease affecting more than 220 million patients worldwide, with approximately 90% having type 2 diabetes (DM2) [1]. Patients with DM2 often have co-morbid affective symptoms such as depression and anxiety. Results of recent studies show that 10-30% of patients with DM2 suffer from major depressive disorder or sub-threshold depression [2-4], about 14% suffers from generalized anxiety disorder and up to 40% has an elevated level of anxiety symptoms [5]. A meta-analysis of longitudinal studies showed that diabetes patients are also at a 24% increased risk of developing depression [6].

The high prevalence of depression and anxiety in patients with DM2 has significant negative implications. It is associated with poorer quality of life, impaired self-care activities, higher health care costs, a higher risk for the development of diabetes complications, and increased mortality rates [7-13]. Despite these known adverse effects and the high prevalence of depression and anxiety in DM2, and the fact that effective treatments are available, there is a considerable underdetection and subsequent undertreatment of these conditions [14,15]. Less than half of the depressed and/or anxious patients with diabetes are recognised as such [14,15]. In order to prevent the negative consequences of anxiety and depression, early detection and enhanced treatment thus seem crucial.

Meta-analyses have shown that treating depression and anxiety in patients with DM2 results in reduced psychological distress, but also in improved glyceamic control [16,17]. For example, the meta-analyses by Ismail et al. showed that psychological interventions resulted in a significant better glyceamic haemoglobin, with an absolute difference of 0.76% (or 76 mmol/mol) [16]. A study by Bogner et al. has shown that the all cause mortality risk decreased when treating depression in primary care patients with diabetes mellitus [7]. However, the study by Bogner has been criticized by Thombs and Ziegelstein [18]. Given the high prevalence of depression and anxiety in patients with DM2, and the fact that these emotional problems are often overlooked and undertreated, while effective treatments are available, current guidelines recommend screening for depression and anxiety [19-21]. A recent randomised controlled trial showed, however, that screening alone did not improve depression outcomes in secondary diabetes care [22]. It seems crucial that screening efforts should be embedded in a managed care approach for depression/anxiety [23].

A large American randomised controlled trial, The Pathways Study, tested the effectiveness of a collaborative care approach consisting of screening, stepped care intervention and collaboration between several health professionals (multidisciplinary team) [24]. The collaborative care approach was more effective in reducing depressive symptoms compared to usual care (z = 2.84, P = 0.004 after 6 months). It was also cost-effective [25]. However, no effect on glyceamic control was found [24]. Another randomised controlled trial (n = 361) has been conducted in the Netherlands in elderly primary care patients with diabetes or COPD and co-morbid depression [26]. The intervention, provided at home by trained nurses, was based on CBT principles and self-management. While the intervention was effective in reducing depressive symptoms (BDI improvement rate OR = 3.22 [1.31 - 7.89]), it was not cost-effective [27].

Most research focused on treating depression, and less research has investigated a treatment for anxiety in patients with DM2. The randomised controlled trials investigating anxiety treatment in patients with diabetes show less consistent results compared to the depression trials; some studies showed a beneficial effect while other did not [5,28].

In the present study, we therefore aim to test the effectiveness of a disease management intervention. It will be tested whether and to what extend the **Di**sease **Ma**nagement intervention for **Co**-morbid **De**pression and **A**nxiety in patients with **DM2**(DiMaCoDeA-DM2) can significantly reduce symptoms of depression and anxiety. Using a randomised controlled trial design, we will compare the new intervention to care as usual. Our primary objective is to investigate the effectiveness of the disease management approach on symptoms of depression and/or anxiety. Our secondary objectives are to investigate whether this approach results in improved quality of life, reduced diabetes-specific emotional distress, improved lifestyle and self care behaviours, and lower health care costs.

## Methods/Design

### Eligibility criteria

Eligible patients are type 2 diabetes mellitus patients, aged 18 or over and with elevated depressive (PHQ-9 score ≥ 7) and/or anxiety symptoms (GAD-7 score ≥ 8; see 'assessment' for more information about the PHQ-9 and GAD-7). Patients will be excluded if they currently receive psychological treatment for their symptoms of depression or anxiety, experience major psychiatric problems, such as schizophrenia and suicidal ideation, are addicted to alcohol, drugs or gambling, are cognitively impaired, or are unable to read or speak Dutch sufficiently.

### Study setting and sample recruitment

The study will be conducted in primary care practices that are affiliated to a large primary care organisation PoZoB (Praktijkondersteuning Zuidoost Brabant). Over 200 general practitioners and approximately 150 practise nurses in a southern region of the Netherlands are associated with PoZoB with approximately 12.000 patients with DM2. The general practitioners, together with the practice nurse are responsible for the primary care of patients with chronic diseases such as DM2. Patients with DM2 are seen by the practice nurse every three months.

Patients with DM2 from the general practices that agreed to participate will be screened for symptoms of depression or anxiety with the PHQ-9 and GAD-7. Eligible patients will be invited for an interview. During this interview the baseline questionnaires will be administered. When eligible patients agree to participate and after they have given written informed consent, they will be randomised into the intervention group or the care as usual group.

### Randomisation

Patients will be randomised on patient level in equal ratio (1:1). Block randomisation will be used with block sizes of 2 and 4. These block sizes are chosen to enhance the chance that in each general practice patients will be in both study conditions. An independent researcher will generate a random sequence by http://randomization.com and will fill envelopes with the sheets describing the group allocation. These opaque envelopes will be sealed and sequentially numbered by the independent researcher. When a participant is enrolled in the study, the person who enrols the participant will open the envelope and disclose the group allocation. The allocation sequence will be concealed until a participant is irreversibly registered.

### Power

Assuming an α of 0.05 and a 1-β (power) of 0.90, 64 participants are needed in each condition to be able to detect a moderate effect of 0.5 standard deviation on the PHQ-9 and GAD-7. We anticipate a drop out of 20% and therefore we will need to include 80 patients in both groups.

### Intervention

**Figure 1:**
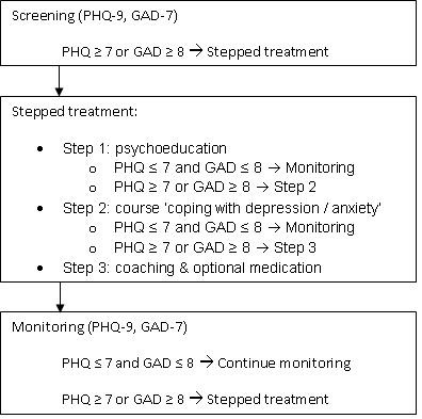
**DiMaCoDeA Intervention**.

The DiMaCoDeA-DM2 intervention will continue for a year and will consist of active screening, stepped care treatment and monitoring of depression/anxiety (see Figure 1).

#### Screening

All patients with diabetes in the collaborating general practices will be screened for symptoms of depression and anxiety using the PHQ-9 and GAD-7.

#### Stepped care

A stepped care model has been used to design the intervention. This means that a basic treatment will be provided firstly, followed by intensified treatment when needed, i.e. in case of non-remission or worsening of symptoms. The DiMaCoDeA-DM2 stepped care treatment will comprise of the following three steps. The first step is psycho-education, which will consist of four 30-minute lessons provided by trained psychologists. At the end of the fourth lesson, the patient will fill out the PHQ-9 and GAD-7. If the patient scores below the cut-off score on both questionnaires (PHQ-9 < 7 and GAD-7 < 8), the treatment will be stopped and the patient will enter a phase in which symptom severity of depression/anxiety will be monitored. If the patient still suffers from significant depression/anxiety, as indicated by scores above the cut-off score, the patient will enter step 2. In the second step the course 'coping with depression and anxiety' will be offered to the patient. The course is based on the "coping with depression" course by Lewinson [29] and a "coping with anxiety" course [30]. The course consists of a self-help book and coaching. Coaching will be provided in the general practice office by trained psychologists and will take place once a week for half an hour. Depending on the most prominent complaints and on the patient's preference either the "coping with depression" course or the "coping with anxiety" course will be provided. A combination of the two courses is also possible. The course takes 10 weeks to complete. Halfway through the course and at the end of the course, the patient will fill out the PHQ-9 and GAD-7. If during the course the symptoms worsen, the patients will be offered the opportunity to start with step 3, even though step 2 has not been finished. In step 3, the GP will offer the patients medication for their symptoms of depression and/or anxiety. If medication is indicated, the general practitioner will have contact with the patient to discuss side effects and monitor the effect of the medication. Moreover, the course will be elongated with a maximum of 6 sessions in six months.

#### Monitoring

A crucial element of the intervention will be the frequent monitoring of depression and anxiety. During the DiMaCoDeA intervention, patients will fill out the PHQ-9 and GAD-7 every three months. If the patient has a score PHQ-9 ≥ 7 or GAD-7 ≥ 8, treatment will be offered; if step 1 has been completed, step 2 will be offered, and if step 2 has been completed, step 3 will be offered.

### Control group

The control group will receive care as usual. During the assessments (see below) the participants will fill out the PHQ-9 and GAD-7. If a patient in the control group has two consecutive PHQ-9 scores ≥ 15 or two consecutive GAD-7 scores ≥ 15, the general practitioner will be notified.

### Assessments

All participants will fill out questionnaires at 7 time points: at baseline, 3, 6, 9, 12, 18 and 24 months. The primary outcomes are symptoms of depression and anxiety as measured by the PHQ-9 and GAD-7 [31,32]. The PHQ-9 is a screening tool that has nine items that correspond to the nine DSM-IV criteria of depression [31]. With the PHQ-9 the patients are asked how often in the last two weeks they were bothered by nine problems such as " Little interest or pleasure in doing things". Each item can be scored from 0 ("not at all") to 3 ("nearly every day"). The total score on the PHQ-9 ranges from 0 to 27. This score indicates the severity of the depressive symptoms; the higher the score, the more severe [31]. A study with primary care patients with diabetes found an optimal cut off score for depression screening of 7 [33]. The cut-off point of 7 on the PHQ-9, was the most optimal cut off score to predict major depressive disorder measured by a diagnostic interview [33]. Therefore the cut-off of PHQ-9 ≥ 7 will be used in this study. The GAD-7 will be used to assess anxiety symptoms. This questionnaire has been developed to assess generalized anxiety disorder, but can also be used as a screener for several anxiety disorders [32]. A cut-off score of 8 has been found to be the most optimal cut-off score, when used as a screener for several anxiety disorders [32]. Therefore, patients with a score GAD-7 ≥ 8 are considered as having significant anxiety symptoms.

During the baseline interview the Mini International Neuropsychiatric Interview (MINI) is administered to assess major depression and general anxiety disorder. The MINI will not be used as a selection criterion.

Secondary outcomes are quality of life, health status, diabetes specific distress, self-management, medication adherence, and cost-effectiveness. Baseline and 12, 24 months HbA1c will be obtained from the patients' charts, to evaluate the effect on blood glucose. Furthermore, we will gather data regarding demographics (age, gender, marital status, and education), exercise (measured with SQUASH [34]), smoking and alcohol use, body mass index, psychiatric history, sleep impairment, and Type D (distressed) personality (DS-14 [35]). Type D is a personality type that is characterised by negative affectivity and social inhibition. Research on Type D personality has been mostly conducted in cardiac patients. In this population, it has been found that persons with Type D personality have a three-fold risk of adverse cardiac outcomes [36].

### Blinding

The nature of the study does not allow blinding of patients, therapists and researchers.

### Statistical analyses

The data will be analysed using intention to treat approach. This means that participants are analysed in the group to which they are allocated, even though the participant did not start the intervention or did not complete the intervention. To test whether the intervention group differs from the control group in terms of demographical and clinical data, T-tests and Chi-square analyses will be used. To test the efficacy of the intervention in achieving favourable outcomes, ANCOVA's will be conducted. The analyses will be adjusted for the possible confounding variables age and sex.

### Ethics

The study has been approved by the medical ethical committee of the Sint Elisabeth Hospital, the Netherlands NL3363.008.10. The trial is registered in the Dutch Trial Register NTR2626.

## Discussion

This randomised controlled trial will test the effectiveness of a disease management model of anxiety and depression symptoms in Dutch patients with DM2 who are treated in primary care. We expect that the managed care intervention will contribute to enhanced treatment of depression/anxiety and a reduction of symptoms of anxiety and depression. We hypothesize that the intervention will also result in improved quality of life, reduced diabetes specific distress, better glycaemic control and lower health care cost. A first strength of the intervention is that it will largely take place in the general practice office. This will make it easier for patients to participate, because they do not have to travel to specialized mental health care institution. A second strength of the intervention is the stepped care approach. This means that the patient receives as much treatment as needed. Providing only the most intensive treatment to specific patients, will reduce the costs of the intervention. A third strength of the intervention is the monitoring of symptoms of anxiety and depression. It is known that anxiety and depressive symptoms often do recur, but are overlooked. It is therefore important to monitor patients to detect recurring anxiety and depressive symptoms and offer treatment if needed. A fourth strength is the RCT design. By randomising patients, possible confounders will be distributed randomly over the groups. Thereby, a possible different outcome between the two groups is most likely to be attributed to the intervention.

A possible limitation in the design is that the GP will be informed when a patient of the control group has two consecutive high scores. As a consequence the GP may start an intervention and this might interfere with care as usual. However, several studies have shown that focusing on detection, does not automatically lead to improved psychological care [22,23,37].

In conclusion, this trial will compare a disease management model with usual care. This model will improve detection of symptoms of depression and anxiety and will provide an easily accessible service to patients to improve their well-being. In the long-term this model might result in less diabetes complications and reduced mortality rate.

## Abbreviations

DM2: Type 2 diabetes mellitus; COPD: Chronic Obstructive Pulmonary Disease; CBT: Cognitive behavioral therapy; BDI: Beck Depression Inventory; OR: Odds ratio; PHQ-9: Patient Health Questionnaire 9 items; GAD-7: General Anxiety Disorder assessment 7 items; SQUASH: Short Questionnaire to Assess Health-Enhancing Physical Activity; DS-14: Type D scale 14 items; ANCOVA: analysis of covariance; RCT: randomized controlled trial; GP: General practitioner.

## Competing interests

The authors declare that they have no competing interests.

## Authors' contributions

FP is the principal investigator. FP and VP designed the study in collaboration with CS and VS. CS drafted the manuscript. FP, VP and VS revised the manuscript critically. All authors have given their final approval of the version to be published.

## Pre-publication history

The pre-publication history for this paper can be accessed here:

http://www.biomedcentral.com/1471-2296/12/139/prepub
